# Thermal tolerance traits of individual corals are widely distributed across the Great Barrier Reef

**DOI:** 10.1098/rspb.2024.0587

**Published:** 2024-09-11

**Authors:** Hugo Denis, Line K. Bay, Véronique J. L. Mocellin, Melissa S. Naugle, Gaël Lecellier, Steven W. Purcell, Véronique Berteaux-Lecellier, Emily J. Howells

**Affiliations:** ^1^ UMR250/9220 ENTROPIE (IRD-CNRS-UR-IFREMER-UNC), Promenade Roger-Laroque, Noumea Cedex, New Caledonia, France; ^2^ ED 129, Sorbonne Université, 4, Place Jussieu, Paris 75252, France; ^3^ National Marine Science Centre, Southern Cross University, Coffs Harbour, New South Wales, Australia; ^4^ Australian Institute of Marine Science, Townsville, Queensland, Australia; ^5^ Institut de Sciences Exactes et Appliquées (ISEA) EA7484, 145, Avenue James Cook, Nouméa BP R4 98 851, New Caledonia

**Keywords:** coral bleaching, coral reefs, acute heat stress, intraspecific variation, heat tolerance, climate warming

## Abstract

Adaptation of reef-building corals to global warming depends upon standing heritable variation in tolerance traits upon which selection can act. Yet limited knowledge exists on heat-tolerance variation among conspecific individuals separated by metres to hundreds of kilometres. Here, we performed standardized acute heat-stress assays to quantify the thermal tolerance traits of 709 colonies of *Acropora spathulata* from 13 reefs spanning 1060 km (9.5° latitude) of the Great Barrier Reef. Thermal thresholds for photochemical efficiency and chlorophyll retention varied considerably among individual colonies both among reefs (approximately 6°C) and within reefs (approximately 3°C). Although tolerance rankings of colonies varied between traits, the most heat-tolerant corals (i.e. top 25% of each trait) were found at virtually all reefs, indicating widespread phenotypic variation. Reef-scale environmental predictors explained 12–62% of trait variation. Corals exposed to high thermal averages and recent thermal stress exhibited the greatest photochemical performance, probably reflecting local adaptation and stress pre-acclimatization, and the lowest chlorophyll retention suggesting stress pre-sensitization. Importantly, heat tolerance relative to local summer temperatures was the greatest on higher latitude reefs suggestive of higher adaptive potential. These results can be used to identify naturally tolerant coral populations and individuals for conservation and restoration applications.

## 1. Introduction

Global warming modifies species’ thermal environments [1] and directly impacts organism fitness [2]. The persistence of species and the ecosystem services they provide, therefore, depend on their response to rising and extreme temperatures. Yet, considerable intraspecific variation in thermal tolerance exists in marine ecosystems [3] as some species have colonized wide geographical areas spanning large thermal gradients [4]. Individuals can adjust to local temperatures within their lifespan through physiological acclimatization and selection for particular phenotypes within populations can lead to genetic adaptation across generations [5]. Documenting phenotypic differences along thermal gradients can thus inform conservation and restoration efforts [6] by improving predictions of species adaptive potential [3], climate refugia [7] or extinctions [8].

Reef-building (scleractinian) corals are among the organisms most at risk from global warming as they occupy narrow thermal niches in environments where current summer temperatures often approach and increasingly exceed their upper thermal limits [9]. The energetic requirements of corals rely on a finely tuned symbiotic relationship with photosynthetic dinoflagellates (family Symbiodiniaceae) [10] that can be severely impaired under heat stress [11], triggering a loss of symbiont cells and/or their photosynthetic pigments known as coral bleaching [12,13]. Increasingly frequent mass bleaching events have been observed over the past four decades [14,15] and are predicted to have impacted >70% of the world’s coral reefs [16], leading to widespread coral mortality [17,18]. Understanding the extent of variation in coral heat tolerance across phylogeny, space and time is, therefore, crucial to predict the future of these ecosystems [19–22].

Intraspecific variation in coral heat tolerance exists at local scales [23–29] and is an indicator of adaptive capacity and metapopulation persistence probability [30,31]. Populations with low intraspecific variation may risk extirpation at local scales [18,32,33]. On the contrary, high intraspecific variation in physiological traits can buffer populations against the loss of genetic and functional diversity [25]. The thermal tolerance traits of individual corals are shaped by the interaction between the environment (i.e. their thermal history [34–36] but also other factors such as irradiance [37], hydrodynamic regimes [38], nutrients [39] and water oxygen content [40]) and a continuum of biological mechanisms including phenotypic plasticity [41,42], heritable genetic effects [28,43,44] and/or differences in symbiont communities [27,45–47]. Yet, further research is required to understand the relative importance of these drivers for current and future responses to marine heatwaves.

Quantifying intraspecific variation in thermal thresholds during natural marine heatwaves is challenged by differences in heat-stress magnitude and duration over space and time and the technical constraints of surveying large spatial areas during a single heatwave event. Consequently, controlled experiments have been widely used to characterize intraspecific variation in thermal tolerance among reefs [48–52], habitats [29,47,53] and individuals [54,55]. Recently, acute heat-stress assays (<48 h) have been developed to measure individual thermal thresholds in a standardized, high-throughput and cost-effective manner [48–51,53,56–60]. Thermal tolerance rankings were shown to yield consistent results across weeks [61], seasons [56] and with long-term heat stress [53,57] although recent studies suggest that response to long-term and short-term heat stress might differ at the individual level [60,62]. In these experiments, coral heat tolerance has been mostly inferred from photochemical efficiency (*F*_v_/*F*_m_), a common proxy of bleaching onset [63]. However, the role of symbiont photochemical damage in bleaching initiation has been increasingly questioned [64,65] and can be offset by host protective mechanisms [12,13,66] stressing the importance of relating *F*_v_/*F*_m_ measurements to subsequent coral bleaching responses (i.e. the loss of symbiont cells and/or chlorophyll). To date, heat-stress assays have investigated the response from a few colonies at many reefs (e.g. [49]) or many colonies at a few reefs (e.g. [54]), but have not yet evaluated intraspecific variation across multiple spatial scales.

To address the aforementioned knowledge gaps, we used acute heat-stress assays to measure photochemical efficiency and bleaching traits in 768 colonies of *Acropora spathulata* from 14 reefs spanning 1060 km (approximately 9.5° latitude) of the Great Barrier Reef (GBR; electronic supplementary material, figure S1*a*). *Acropora spathulata* is an abundant corymbose species found on reef flats and upper slopes that builds ecologically important structural complexity on the GBR (electronic supplementary material, figure S1*d*) [67]. We identify reefs where heat-tolerant individuals are most likely to be found and use multiple environmental datasets from satellite observations and numerical models to determine environmental predictors of tolerance traits. The large sample size of this study provides GBR-wide information to inform conventional management via spatial and temporal protection and novel interventions, including seeding heat-tolerant corals onto reefs.

## 2. Results

Acute heat-stress assays were performed using standardized temperature treatments to elicit increasing levels of heat stress of 0°C (control), 3, 6 and 9°C above the local maximum monthly mean (MMM) [68] (electronic supplementary material, figure S1*b*). Following exposure of replicate fragments of *A. spathulata* colonies to each treatment, two phenotypic traits were measured: the maximum photochemical efficiency of photosystem II (*F*_v_/*F*_m_) and the hyperspectral imagery estimate of chlorophyll content (normalized difference vegetation index; NDVI; electronic supplementary material, figure S1*c*). As experimental treatments did not reach target temperatures at St Crispin Reef, this site was removed from further analyses resulting in a total number of 709 colonies (electronic supplementary material, table S2). The decline of each colony trait was modelled over temperature treatments using dose–response curves and was used to calculate two colony-level metrics of heat tolerance: the temperature that resulted in a 50% decline in response (i.e. the median effective dose, or ED50) which represents an absolute thermal threshold, and the performance under extreme heat (+9°C), representing how coral traits decline relative to their local reef MMM.

**Figure 1.**
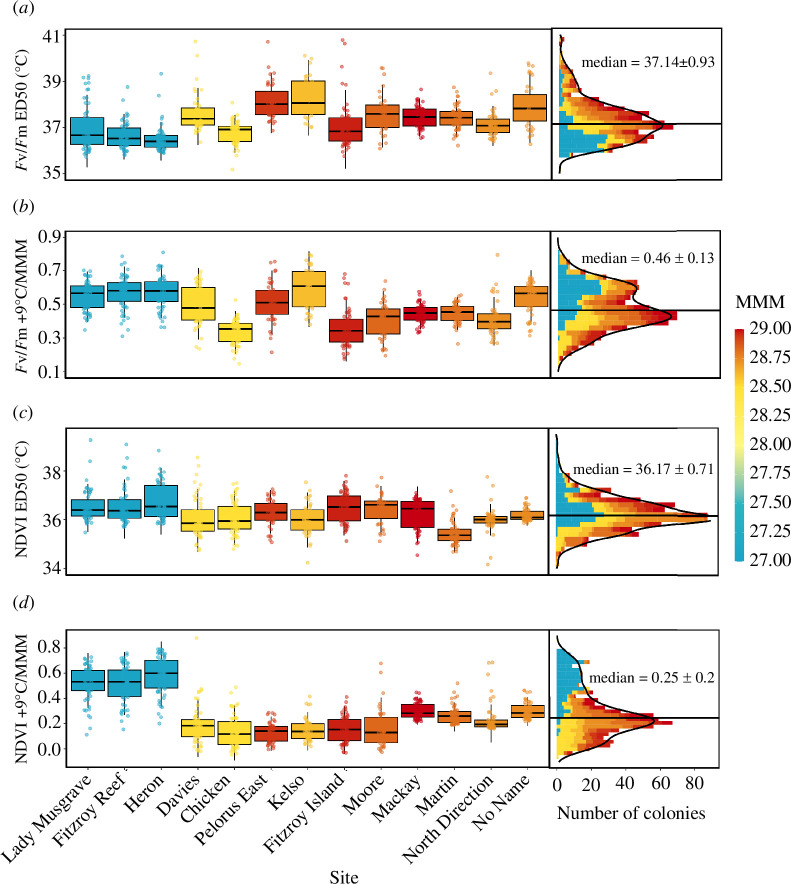
Variation in *Acropora spathulata* thermal tolerance among 14 reefs on the Great Barrier Reef (GBR) under acute experimental heat stress. The decline in maximum quantum yield of photosystem II (*F*_v_/*F*_m_) and total chlorophyll content (NDVI) was used to measure ED50 temperatures (*a,c*) and performance retention under extreme heat (i.e. + 9°C/MMM; *b,d*). The left panels display boxplots of the median (solid line) and the first and third quartiles (box) for each reef coloured by their MMM. The right panels show the distribution of values, with the GBR-wide median and standard deviation. Reefs are ordered (left to right) by increasing latitude.

The majority of *A. spathulata* colonies on the GBR showed a minimal decline in photochemical efficiency (*F*_v_ */F*_m_) and chlorophyll content (NDVI) at +3°C (average ≤5.5%) and +6°C (average ≤7%) above their local MMM. However, under the more extreme temperature of +9°C, these traits declined by 2−80% (*F*_v_ */F*_m_) and 10−98% (NDVI), driving differences in thermal threshold (ED50) and performance retention metrics among reefs and individuals (figure 1).

### (a) Reef-level variation in coral heat tolerance

The GBR-wide medians of colony thermal thresholds were 37.1 ± 0.9°C for *F*_v_ */F*_m_ ED50 (median ± s.d.) and 36.2 ± 0.7°C for NDVI ED50, with reef accounting for 34 and 25% of their respective total variation (ANOVA ω^2^ effect sizes; electronic supplementary material, table S5b). Mean reef-level *F*_v_ */F*_*m*_ ED50s differed by 1.8°C and generally decreased with latitude (figure 1*a*). For example, *F*_v_ */F*_m_ ED50 thresholds in the southern GBR (Heron Island and Fitzroy Reef; 36.5−36.6°C) were 0.8°C lower than all reefs in the central and northern GBR (36.8−38.3°C; *p.adj* < 0.0001; *post-hoc* Games Howell test; electronic supplementary material, table S5c). However, several reefs had higher (Kelso Reef, Pelorus Island, No Name Reef) or lower (Fitzroy Island, North Direction Island) ED50s than nearby reefs at similar latitudes (*p.adj* < 0.05). Conversely, NDVI ED50 thresholds were less variable among reefs (by 1.3°C) and were lower at Martin Reef in the northern GBR than all other reefs (*p.adj* < 0.01; Figure 1*c*).

**Figure 2.**
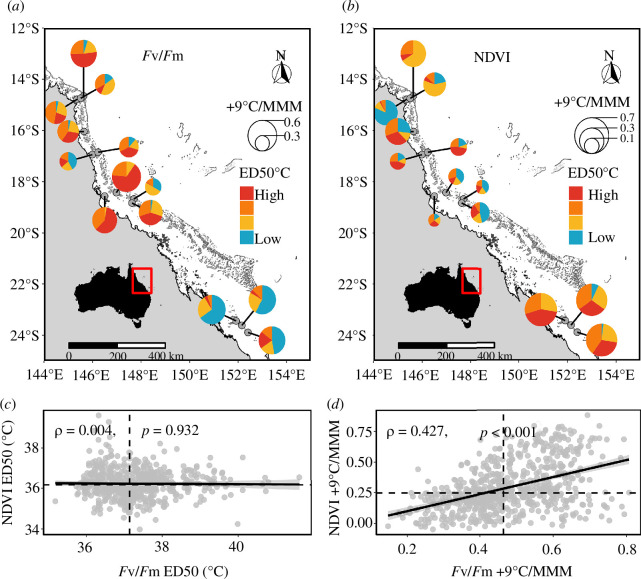
Spatial distribution of *Acropora spathulata* acute heat tolerance on the Great Barrier Reef measured by (*a*) maximum quantum yield of photosystem II (*F*_v_/*F*_m_) and (*b*) chlorophyll content (NDVI). Pie charts at each reef represent the proportion of individual colony ED50s falling between each quartile of the global distribution (blue: ED50 < Q1, yellow: Q1 < ED50 < Q2, orange: Q2 < ED50 < Q3, red: Q3 < ED50). Pie chart radii are proportional to average retained performance under extreme heat (i.e. +9°C/MMM), normalized by the global range for plotting purposes. Correlation between (*c*) *F*_v_/*F*_m_ and NDVI ED50s and (*d*) retained performance under extreme heat (+9°C/MMM). Each point represents a distinct colony (*n* = 586 and 675 for (*c*) and (*d*), respectively) and Spearman’s-ρ and *p*-values are indicated.

GBR-wide performance retention (performance under +9°C relative to local conditions) was 48 ± 12% for *F*_v_ */F*_m_ and 29 ± 19% for NDVI with reef accounting for 44 and 59% of their respective total variation (ω^2^ effect sizes; electronic supplementary material, table S5b). Performance retention was 2−41% (*F*_v_ */F*_m_) and 60−65% (NDVI) higher at southern (higher latitude) reefs than the rest of the GBR (figure 2*b,d*), thus following an opposite trend to *F*_v_ */F*_m_ ED50.

### (b) Colony-level variation in coral heat tolerance

At the colony level, thermal thresholds varied by up to 6°C, approximately three times the reef-level variation (1.8°C range; electronic supplementary material, table S5a). Within reefs, there was a 3.1°C and 2.5°C variation in colony-level ED50s for *F*_v_ */F*_m_ and NDVI, respectively (95% range; electronic supplementary material, figure S2). The magnitude of this variation differed among reefs (*p* < 0.001, Levene’s test; electronic supplementary material, table S5b) but no specific reef exhibited higher or lower variation for both traits. Colony-level variation in performance retention under +9°C was also higher than reef-level variation (by 48% for *F*_v_ */F*_m_ and by 17% for NDVI). Although colony-level ED50s were prone to uncertainty (median 95% CI range = 2.51 ± 3.34°C and 2.99 ± 5.56°C; electronic supplementary material, Material and methods), congruent patterns between ED50 and performance retention support high variation in acute heat tolerance among and within reefs of the GBR.

**Figure 3.**
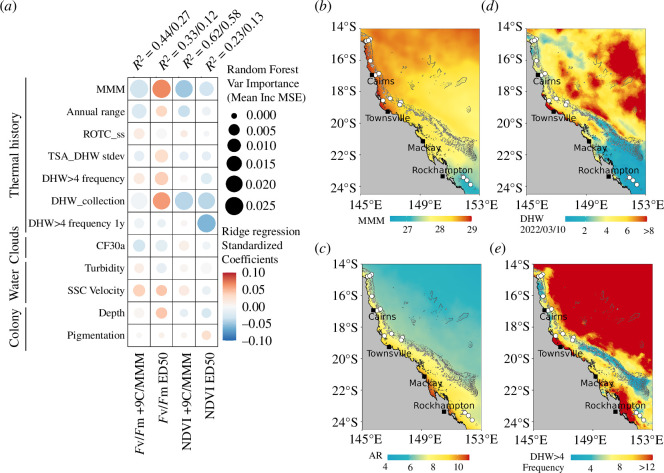
Environmental drivers of acute heat tolerance of *Acropora spathulata* on the Great Barrier Reef. Heat tolerance was measured as the maximum quantum yield of photosystem II (*F*_v_/*F*_m_) and chlorophyll content (NDVI) expressed as ED50 temperature thresholds and performance retention under extreme heat (+9°C). Relationships between environmental predictors (defined in electronic supplementary material, table S7) and heat-tolerance metrics were assessed using ridge regression and random forest models. (*a*) Heatmap of associations evaluated with random forest variable importance (10-fold stratified cross-validation, circle size) and ridge regression standardized coefficients (multiple bootstrap resampling *n* = 100, circle colour). The variance explained by each model (random forest/ridge R^2^) is shown above each tolerance trait metric. MMM = maximum monthly mean (1985–1990+1993 climatology), ROTC_ss = rate of temperature change in spring/summer, TSA_DHW_s.d. = standard deviation of degree heating weeks (DHW), DHW_collection = DHW at the day of collection, DHW > 4 frequency 1 y = frequency of DHW > 4 in the year prior to collection, CF30a = cloud fraction anomaly, the difference between the average cloud fraction for 30 days prior to collection and the long-term average cloud fraction for that location (2002−2022), SSC Velocity = average sea surface currents velocity. (*b*–*e*) Thermal history maps of (*b*) maximum monthly mean temperature as defined by NOAA [68], (*c*) annual temperature range (2014−2022), (*d*) peak degree heating weeks during sampling and (*e*) the annual frequency of degree heating weeks above four since 1985. White dots indicate reef sites sampled in this study.

Importantly, heat-tolerant colonies were widely distributed across the GBR. For example, colonies that were ranked within the top 25% of *F*_v_/*F*_m_ ED50 and NDVI ED50 occurred at virtually all reefs (figure 3*a,b*). However, the most tolerant colonies were generally more abundant at central and northern reefs for *F*_v_/*F*_m_ and southern reefs for NDVI. Additionally, several reefs exhibited a high proportion of tolerant individuals for both traits (e.g. Mackay Reef, Moore Reef and Pelorus Island; Figure 2).

### (c) Response to acute heat stress differs between traits

The ranking of reef- and colony-level responses under heat stress differed between phenotypic traits. Overall, there was no GBR-wide correlation between *F*_v_ */F*_m_ and NDVI ED50s (Spearman’s-*ρ* = 0.00, figure 2*c*), but a moderate positive correlation between their performance retention at +9°C (*ρ* = 0.43, figure 2*d*). At the reef level, there was a moderate positive correlation between *F*_v_ */F*_m_ ED50 and NDVI ED50 at Chicken and No Name (*ρ* > 0.3) and a weak positive correlation at Davies, Heron, Mackay, Martin and Moore (*ρ* = 0.1−0.3; electronic supplementary material, table S6). Similarly, there was a weak to moderate positive correlation between the performance retention of *F*_v_ */F*_m_ and NDVI at all reefs (*ρ* = 0.1−0.3) except at Chicken (*ρ* = 0.02), North Direction (*ρ* = 0.84) and Pelorus Island (*ρ* = −0.22; electronic supplementary material, table S6).

### (d) Environmental drivers of acute heat-tolerance metrics

To examine environmental conditions experienced by *A. spathulata* across the GBR, we retrieved 10 environmental variables characterized by 24 quantitative predictors computed from *in situ* loggers, numerical models and satellite observations (electronic supplementary material, table S7). A principal component analysis (PCA) on environmental predictors divided the 709 colonies into three major clusters (electronic supplementary material, figure S3). The first component (46.4% explained variance) separated cooler southern reefs from warmer central and northern (lower-latitude) sites and was mainly driven by MMM (1.7°C range) and degree heating weeks (DHW) at the time of collection. The second component (14.7% explained variance) separated northern and central sites along an inshore–offshore gradient that was mainly driven by turbidity, sea surface current velocities and temperature variation (annual range and the rate of change from spring to summer). Within clusters, colonies grouped by reefs as most predictors were retrieved at the site level and were minimally separated by their water depth or pigmentation. Pigmentation scores prior to collection differed between individuals and sites but had no clear association with DHW at the time of collection (electronic supplementary material, figure S4).

Environmental drivers of heat-tolerance traits (ED50 and performance retention in *F*_v_/*F*_m_ and NDVI) were evaluated using random forest (RF) ensemble learning and ridge regression (RR) on 12 low to moderately correlated predictors (pairwise absolute Pearson correlation = 0.01−0.69; electronic supplementary material, figure S5). Together, these predictors explained a low to moderate proportion of variation in heat stress responses of *A. spathulata* across the GBR (*R* ^2^ = 0.12−0.62). The predictive accuracy of RF and RR was stronger when heat tolerance was expressed for both *F*_v_/*F*_m_ and NDVI as performance retention (*R* ^2^ = 0.27−0.62) than ED50 thresholds (*R* ^2^ = 0.12−0.31). Top environmental predictors slightly differed among traits and metrics but variation in heat tolerance was primarily associated with site-level thermal history (figure 3). MMM (figure 3*b*) and DHW at the time of collection (figure 3*d*) were predominant metrics associated with response to acute heat stress (i.e. top RF predictors for three of the four heat-tolerance metrics; electronic supplementary material, table S9a) while being strongly correlated to each other (*R* = 0.78). The direction of associations differed between traits with MMM and DHW at the time of collection being positively associated with *F*_v_/*F*_m_ ED50 (β = 0.06−0.07), but negatively with performance retention in *F*_v_/*F*_m_ and NDVI and NDVI ED50 (β = −0.03 to −0.06; electronic supplementary material, table S9b). Secondary thermal history associations occurred between the annual range in temperature and both performance retention metrics (*F*_v_/*F*_m_ β = −0.03, NDVI *β* = −0.04; Figure 3*c*), and between the frequency of DHW > 4 and both ED50 thresholds (*F*_v_/*F*_m_ ED50 *β* = 0.04, NDVI ED50 *β* = −0.06; Figure 3*e*). Particularly, the frequency of DHW > 4 in the year before collection showed a strong negative association with NDVI ED50 (β = −0.060, top predictor in RF), but no significant association with any other trait metric.

To a lesser extent, the heat tolerance of *A. spathulata* was associated with water flow, shading and depth. *F*_v_ */F*_m_ tolerance metrics were generally higher at reefs with high sea surface current velocity (*F*_v_ */F_m_* ED50: *β* = 0.042, +9/MMM: *β* = 0.037) and lower at sites with positive cloud anomalies in the 30 days prior to collection (*F_v_/F_m_* ED50: *β* = −0.017, +9/MMM: *β* = −0.031; Figure 3*a*). Turbidity showed little to no relationship with heat-tolerance metrics. At the colony level, increasing water depth was positively related to *F*_v_ */F*_m_ metrics (*F*_v_ */F*_m_ ED50: *β* = 0.042, +9/MMM: *β* = 0.004) but negatively related to NDVI metrics (NDVI ED50: *β* = −0.028, +9/MMM: *β* = −0.015). In addition, colonies with higher levels of pigmentation at the time of collection tended to have higher NDVI ED50 thresholds (*β* = 0.028), but there was no relationship with other tolerance metrics.

## 3. Discussion

The large geographic scope of this study (13 reefs across 9.5° latitude) and sample size (*n* = 709) revealed extensive intraspecific variation in the heat tolerance of reef-building corals across the GBR. This resulted in more than 6°C variation in colony thermal thresholds (figure 1; electronic supplementary material, table S5a) where tolerant colonies—based on multiple traits and metrics—were widely distributed among and within reefs. Environmental predictors of heat-tolerance variation support adaptation and/or acclimatization of populations to local conditions while unexplained differences among colonies may be due to host and/or symbiont adaptive genetic variation within reefs.

### (a) Acute heat-tolerance metrics linked to historical and recent environmental conditions

All heat-tolerance metrics were most strongly related to thermal history supporting adaptation and/or acclimatization of corals to their local thermal regimes (figure 3*a*) [35,36,69,70]. Heat-tolerance metrics first varied along a latitudinal gradient, reflecting differences in average temperatures. For instance, *F*_v_/*F*_m_ ED50 thresholds increased at reefs exposed to higher summer temperatures (MMM, figure 3*b*), which corroborates ecological experiments that found a 4°C difference in *A. spathulata* larvae survival thresholds between Lizard Island (14.7 °S) and One Tree Island (23.5 °S); i.e. the same latitudinal range as our study [71]. Tolerance metrics also varied by shelf position, with higher *F*_v_/*F*_m_ ED50 thresholds at reefs subjected to large annual thermal variation (AR, figure 3*c*), and by exposure to marine heatwaves (frequency of DHW > 4 and standard deviation of DHW, figure 3*e*). *F*_v_/*F*_m_ ED50 also increased with the accumulated heat stress (DHW) at the time of sampling (figure 3*d*), supporting that acclimatization of the holobiont to moderate heat stress (0−4°C week^−1^), possibly through upregulation of photoprotective mechanisms, can increase the heat-tolerance trait *F*_v_/*F*_m_ ED50 as reported by Cunning *et al.* [56]. However, *F*_v_/*F*_m_ ED50 was lower at reefs where accumulated heat stress was the highest (Chicken and Davies; approx. 4.6°C week^−1^) suggesting that higher levels of prior heat exposure may be detrimental [42]. As such, we observed weaker unexpected negative associations between NDVI ED50 and thermal history metrics, notably with the frequency of DHW > 4 in the 12 months prior to sampling. This is similar to *F*_v_/*F*_m_ ED50 findings of Marzonie *et al*. [49] in the Coral Sea, suggesting that some reef populations may be impacted over long time periods after heat exposure. Differences in the association with thermal history among traits could thus occur from a decoupling between host and symbiont baseline conditions and/or stress responses (discussed below) [64–66]. Nevertheless, several reefs in our study (No Name, North Direction, Mackay) exhibited a high proportion of tolerant individuals for both *F*_v_/*F*_m_ and NDVI ED50, showing that some colonies and populations may perform better under heat stress across multiple traits [72]. These populations will be more likely to play a key role in the resilience of the GBR in a warming climate, and thus their mapping in space and time could provide valuable information for spatial planning of Australia’s marine parks into the future.

The heat-tolerance metrics of *A. spathulata* measured here were further associated with predictors that influence water movement, light and within-reef temperature (figure 3*a*). Strong water movement can delay photochemical damage under thermal stress through coral surface cooling, increases in respiration and metabolic transfers and removal of toxins [38,73], and the positive association between surface current velocity and *F*_v_/*F*_m_ metrics suggests a carryover of these effects to our experiments. Solar irradiance is also a factor known to mediate bleaching responses under high seawater temperatures [74] and *F*_v_/*F*_m_ ED50 thresholds decreased at reefs exposed to low irradiance in the weeks preceding collection (i.e. high cloud cover). This may indicate that symbionts acclimated to lower light levels [75] experienced greater photodamage under the irradiance of the experimental system [76]. These results demonstrate that empirically derived thermal tolerance metrics are not only shaped by long-term adaptation but also acclimatization to recent conditions. Overall, the proportion of variation explained by reef-level environmental predictors was low to moderate (*R* ^2^ = 12–62%), highlighting the extent of heat-tolerance variation at local scales.

### (b) Standing within-reef variation in heat tolerance supports adaptive potential to climate change

There was greater variation in the acute heat tolerance of *A. spathulata* colonies within reefs than among reef-level averages (ED50 3.1 versus 1.8°C and 2.5 versus 1.6°C for *F*_v_/*F*_m_ and NDVI, respectively; electronic supplementary material, table S5a), despite most colonies being sampled from a narrow depth range at a single site less than 600 m^2^. Importantly, we found that heat-tolerant corals (defined as the top 25 percentile from the GBR ED50 distributions) occurred at nearly every reef, including the cooler southern GBR. These results align with previous results on *A. cervicornis* in the Florida Keys [56], *A. hyacinthus* in Palau [51] and *A. hyacinthus* in the GBR [77]. Within-reef variation remained mostly unexplained by colony-level predictors (depth and pigmentation) and could be elucidated by incorporating genomic data not yet available here. Individual differences in heat tolerance may be underpinned by genomic variation within the host [23,28], symbionts [45,78] and/or their interactions [55]. For example, incorporating the proportion of *Durusdinium* cells and host polygenic scores based on putative heat-adaptive loci increased the predictive accuracy of *A. millepora* bleaching models on the GBR by approximately 22% [79]. In addition, deciphering the independent role of environmental drivers requires a better characterization across microhabitats [80] and depth [81], as well as optimization of sampling strategies across environmental gradients to reduce the collinearity of predictors [82]. The large standing variation in thermal tolerance at the reef scale reported in this study has important implications for the conservation of this species on the GBR. It may notably support *A. spathulata* adaptation to climate change through the selection of pre-existing heat-tolerance alleles [20]. Furthermore, rather than translocating individuals from warmer reefs (e.g. Howells *et al*. [69]) restoration projects can target local heat-tolerant individuals when their phenotypes are known or by sampling a diversity of genets.

### (c) Coral populations from the southern GBR may live further from their upper thermal limit

Acute thermal thresholds (ED50) for *A. spathulata* populations on the GBR were on average 9.5−9.6°C above their local MMM on southern reefs compared with 7.5−8.8°C on central and northern reefs (electronic supplementary material, table S5a). This is consistent with higher performance retention under extreme heat on southern reefs and latitudinal differences in the thermal thresholds of *A. spathulata* larvae [83]. Consequently, under comparable levels of acute thermal stress, southern populations might be less susceptible to bleaching than central and northern populations. In line with this expectation, southern GBR reefs have experienced less frequent and intense bleaching events than central and northern reefs over the 1985−2022 period [84] while undergoing similar maximum DHW across the 2016, 2017 and 2020 major bleaching events [15]. The lower frequency and magnitude of bleaching events on southern reefs might also allow more time for adaptation to occur through the migration of heat-tolerance-associated alleles from warmer northern reefs [20].

Conversely, the low cumulative heat stress experienced by those regions may result in coral assemblages that are naive to high heat stress [15]. As such, southern reefs underwent mild but highly prevalent coral bleaching in 2020 (86% of colonies; Page *et al.* [85]; 48% of colonies; Nolan *et al*. [86]) and the probability of severe bleaching was seven times higher than for central and northern reefs under the same cumulative heat stress (DHW) [15]. Further investigations are thus required to understand how recurrent warming disturbances and gene flow with northern regions will shape the evolution of these cooler reefs in natural conditions.

### (d) Divergent effects of acute heat stress on thermal tolerance traits

Here we found a moderate correlation between *F*_v_/*F*_m_ and NDVI performance retention under extreme heat (figure 2*c*). This supports the use of photochemical apparatus integrity as an early proxy of coral bleaching [53,57,63] and the importance of symbiont tolerance in bleaching response. For instance, some symbiont species or genotypes may produce more photoprotective pigments or antioxidant compounds, alleviating oxidative stress for the host [12,13]. Conversely, we found no correlation between ED50s of *F*_v_/*F*_m_ and NDVI traits (figure 2*d*). The only two other studies using *F*_v_/*F*_m_ and chlorophyll-related ED50 thresholds found congruent reef-level variation between the two traits for some species (e.g. *Pocillopora verrucosa* [48] and *A. hyacinthus* [77] but not others (e.g. *Porites lobata* [48]). In both studies, visual chlorophyll scores showed higher noise and lower differences in reef ED50s, which align with *A. spathulata* pairwise reef differences in NDVI ED50s (0.39°C) being almost half that of *F*_v_/*F*_m_ ED50 (0.67°C). This may also be due to differences in the timing of measurement between traits as their decline occurs at different rates [64]. In *A. tenuis*, *F*_v_/*F*_m_ values were found to be stable for 0−24 h after the end of an acute heat stress, while chlorophyll decreased up to 24 h after the end of the temperature ramp-down [87]. Therefore, delaying the timing of NDVI measurements in our study may have revealed higher divergence in chlorophyll retention between reefs. Finally, algal oxidative stress and photosynthetic damage can be alleviated by host processes [13,66] and thus occur without a decrease in chlorophyll content or symbiont density [88].

Divergent responses between traits highlight the importance of defining heat tolerance and selecting phenotypic traits accordingly. Chlorophyll content may be an appropriate proxy for holobiont bleaching and mortality as its decline during acute heat stress can persist and reflect mortality in the next month [61]. However, photochemical efficiency may be a better proxy to detect sublethal effects as corals can experience physiological stress of reduced tissue biomass and symbiont loss long before changes in pigmentation are visually detectable [64]. Finally, which phenotypic trait would best predict heat tolerance in natural conditions remains unclear as colony responses can vary between acute and long-term heat stress [60,62]. To validate the ecological relevance of acute heat-stress assays, future studies should compare these rankings with natural bleaching observations, while accounting for the difference of heat stress exposure among reefs under natural marine heatwaves. Our results demonstrate that no single trait or metric can fully capture the complexity of the coral holobiont heat-stress response, and highlight the importance of measuring multiple traits whenever achievable, including relevant host traits (e.g. antioxidant capacity).

## 4. Conclusion

Using standardized acute heat-stress assays, we found that heat-tolerant corals were widely distributed across the GBR, including cooler, southern reefs. This suggests potential for adaptive responses to climate change, if heat-tolerance traits have a heritable basis [31,44]. Rankings of coral heat tolerance can guide human interventions and their interpretation and application may depend on the specific traits and metrics measured. Corals with high absolute thermal tolerance can help to decipher heat stress protective mechanisms [23,89] and be used as material for assisted gene flow through translocation [59,90] and selective breeding [43]. On the contrary, corals with high thermal tolerance relative to local conditions may be better targets for local propagation and breeding, decreasing the risk of carrying pathogens and phenotype-environment mismatches [91]. Nonetheless, natural adaptive processes and human interventions must be accompanied by reduced anthropogenic emissions through ambitious national and international commitments to secure the persistence of species and populations across the entire GBR.

## 5. Methods

### (a) Study sites, experimental design and set-up

#### (i) Study sites and sample collection

We sampled 768 colonies of *A. spathulata* across 14 reefs of the GBR (electronic supplementary material, figure S1*a* and table S1). At each reef, 40−60 colonies were sampled on SCUBA from one or two sites (covering 50−4200 m^2^) between 28 February and 26 March 2022 (GBRMPA permit G21/45166.1) over a single or two consecutive days. Colonies were sampled from the upper reef slope to reef flat over a total depth range of 5 m across the GBR, and we avoided sampling neighbouring colonies of identical pigmentation to reduce the likelihood of sampling clonemates. For each colony, 12 nubbins (approximately 7 cm) were collected for standardized acute heat-stress experiments and *in situ* metadata was recorded. This included colony-level GPS coordinates linked to *in situ* photographic records [92], time-corrected depth (average = 0.51 ± 1.3 m relative to the lowest astronomical tide) and visual pigmentation (coral watch health chart scores at 0.5 intervals) [93]. Colonies showing signs of disease were rarely encountered but intentionally excluded to avoid physiological bias unrelated to temperature effects.

#### (ii) Acute heat-stress assay design

A portable automated experimental system (‘Seasim-in-a-box’, National Sea Simulator, Australian Institute of Marine Science; electronic supplementary material, figure S6) was modified from Marzonie *et al*. [49] to conduct acute heat-stress assays onboard a research vessel the day following collection (electronic supplementary material, Material and methods). The experimental design consisted of three replicate tanks for each of four temperature treatments of 0 (control), 3, 6 and 9°C above the local MMM, 1985–1990+1993 climatology as defined by NOAA [68] for each reef site. These treatments were designed to elicit increasing levels of thermal stress following Voolstra *et al.* [53]. Immediately after collection, 12 nubbins from each coral colony were mounted on separate experimental racks with unique identifiers and held overnight at ambient seawater temperature. The following morning, one nubbin from each colony was randomly assigned to each of the 12 tanks electronic supplementary material, figure S1*b*). Assays started at 11.00 in the morning and consisted of 3 h ramp-up from ambient temperature (within 1°C of the MMM) to target temperature, 3 h hold at the target temperature, 2 h ramp-down to MMM and a final approximately 12 h hold at MMM (electronic supplementary material, figure S1*c*). During the experiments, temperatures in individual tanks were recorded at 1 min intervals using HOBO loggers (Onset) and closely matched their target profiles (mean_∆*T*_ = 0.36°C; electronic supplementary material, table S2) with the exception of one site which was removed from further analyses (St Crispin: mean_∆*T*_ = 1.61°C).

### (b) Phenotypic traits

#### (i) Photochemical efficiency

The maximum quantum yield of photosystem II (*F*_v_ */F*_m_) of coral photosymbionts (Symbiodiniaceae) was used as an initial rapid, non-invasive measure of heat tolerance. A decline in *F*_v_ */F*_m_ reflects early physiological impairment in corals and has been reported to respond consistently between acute and long-term heat exposures [53,57]; but see Klepac *et al.*[60] and Humanes *et al.* [62]. Measurements were taken 2 h after the end of temperature ramp-down ( >1 h dark adaptation) using an Imaging PAM chlorophyll fluorometer (IMAG-K7, Walz Germany) with the following settings: measuring light = 2 (freq = 1), saturation pulse = 7 (int = 30 s), damp = 1, gain = 1. For each fragment, three measurements were extracted from non-overlapping areas and underwent several quality checks and filtration steps (electronic supplementary material, Material and methods). After filtration, a total of 709 colonies and 7847 fragments were retained in further analyses.

#### (ii) Hyperspectral image-based assessment of chlorophyll content

Non-invasive estimates of chlorophyll content were used as a second metric of heat tolerance. The loss of chlorophyll underpins visual bleaching scores commonly used in acute heat-stress experiments and has been shown to correspond with mortality risk [61]. The total chlorophyll content was assessed in fragments from reflectance measurements taken 11 h after the end of the temperature ramp-down using a hyperspectral camera (Resonon, Pika XC2) with the following settings: integration time = 39.2 ms, gain = 0, frame rate = 22 fps. MATLAB software was used to compute the normalized difference vegetation index (NDVI; electronic supplementary material, Material and methods) from reflectance measurements, where NDVI = (*R*_720_− *R*_670_)/(*R*_720_+ *R*_670_) and *R*_*x*_ is the reflectance at *x* nm. NDVI is a proxy for chlorophyll used in a wide range of organisms including scleractinian corals [94] and has been validated in the soft coral *Sarcophton* cf. *glaucum* [95]. We repeated this validation in *A. spathulata* using spectrophotometric determination from tissue extractions [96] with a strong relationship between NDVI and log-transformed chlorophyll-a (*R* ^2^ = 0.74; electronic supplementary material, Material and methods, figure S7).

### (c) Phenotypic data analysis

All statistical analyses were conducted using R v. 4.0.4 and figures created using package *ggplot2* and Inkscape v. 1.2. The response of raw *F*_v_ */F*_m_ and NDVI measurements to temperature across experiments was first assessed with a linear mixed-effect model using the package *lme4* [97] (electronic supplementary material, Materials and methods). The model results confirmed that the tank had a minor effect on phenotypic measurements (explained by 1.7% of *F*_v_ */F*_m_ random variance σ_i_ 2 and 0.8% of NDVI σ_i_^2^; electronic supplementary material, table S3). Likewise, the coral fragment size effect was negligible (*B* = 1.2 × 10^−6^ for *F*_v_ */F*_m_ and *B* = 8.5 × 10^−6^ for NDVI; electronic supplementary material, table S3). These factors were thus discarded from further analyses and phenotypic measurements were used to compute metrics of heat tolerance.

#### (i) ED50 thermal thresholds

For each colony, the decrease in *F*_v_ */F*_m_ and NDVI was modelled over temperature treatments using dose–response curves in the R package *drc* [98]. Colonies with <2 fragments per treatment after previous quality control filtering (electronic supplementary material, Material and methods) were excluded to ensure robust estimates of individual responses across treatments (excluded colonies: *F*_v_ */F*_m_ = 94, NDVI = 4). All models were fitted using the *drm* function based on the mean hold temperature recorded within each tank for each experiment, with constraints set on parameters (electronic supplementary material, table S4a). The best model (Weibull type II with three parameters) was chosen as the one with the lowest Akaike’s information criterion score for most reef sites using the *mselect* function (6/13 and 11/13 reefs for *F*_v_ */F*_m_ and NDVI, respectively; electronic supplementary material, table S4b). Because using the same model is a prerequisite to compare ED50s, we used this model for all colonies even though some curves showed a better fit to log-logistic or quadratic models. The model equation is

,$$f\left(x;b,d,e\right)=d(1-exp(-\exp\left(b\left(\log\left(x\right)-\log\left(e\right)\right)\right))$$

where *b* is the steepness of the curve, *d* is the upper asymptote (lower asymptote set to 0) and *e* is the inflexion point. An example of dose–response curves can be found in electronic supplementary material, figure S8. Dose–response curves were filtered to remove individuals that showed poor fit to the data, notably when the decline in phenotypic traits was minor or absent up to +6°C or +9°C resulting in ED50s with wide confidence intervals (electronic supplementary material, Material and methods) which may have filtered out some of the most heat-tolerant individuals. After filtration 615 and 675 colony-level ED50 estimates were retained for *F*_v_ */F*_m_ and NDVI, respectively.

#### (ii) Performance retention under thermal stress

Performance retention under extreme heat was computed as


$$Xi_{MMM+9/MMM}\;=\;\left(Xi_{MMM+9}/Xi_{MMM}\right)x\;\left(\left(T{mean}_{MMM+9^{\circ }C}-\;T{mean}_{MMM}\right)/9\right)$$

where Xi is the trait (*F*_v_ */F*_m_ or NDVI) averaged across fragments of individual i in the MMM and MMM + 9 treatments, respectively, and *T*mean_MMM+9°C_ and *T*mean_MMM_ are the average hold temperatures for these treatments. The second term accounts for small differences between target and effective temperatures across tanks and experiments. Performance retention can be computed even for colonies that experienced a minor decline in phenotypic traits under +9°C, which prevented fitting a dose–response curve. After filtration of colonies with <2 fragments in the MMM and MMM + 9°C treatments, 675 and 709 colony-level performance retention estimates were calculated for *F*_v_ */F*_m_ and NDVI, respectively.

### (d) Heat-tolerance variation analysis

The variation in heat tolerance (ED50 and performance retention) was first investigated among sites (*Reef-level variation in coral heat tolerance*; electronic supplementary material, table S5a). The homogeneity of variance between sites was assessed using Levene’s test on residuals from group medians (electronic supplementary material, table S5b). Since variance was unequal across sites, we used one-way Welch’s ANOVA to compare site means and Games–Howell *post-hoc* tests with adjusted *p*-values at α = 0.05 for pairwise comparisons (electronic supplementary material, table S5c).

The variation in heat-tolerance metrics was then quantified among individuals across and within sites (*Colony-level variation in coral heat tolerance*). Following Cunning *et al.* [56], we removed the site effect by computing colony-adjusted ED50 as the grand mean of the total distribution plus residuals from their site of origin and used those adjusted ED50 to assess the range of within-site variation (electronic supplementary material, figure S2).

Finally, we investigated the similarity of colony thermal tolerance rankings between *F*_v_ */F*_m_ and NDVI traits using Spearman’s rank correlation as the assumption of normality was violated (*Response to acute heat stress differs between traits*). For both ED50 and performance retention, we computed both GBR-wide and site-level correlations (figure 3*c,d*; electronic supplementary material, table S6).

### (e) Environmental data acquisition and analysis

To examine environmental conditions experienced by *A. spathulata* across the GBR, we retrieved 10 environmental variables characterized by 24 predictors computed from *in situ* observations, numerical models and satellite observations (electronic supplementary material, Material and methods, table S7). In addition to environmental variables at the reef level, we included colony-level depth obtained from dive computers and adjusted to the tide at the time of collection (standardized to the lowest astronomical tide; electronic supplementary material, figure S9). We also included pigmentation prior to collection to account for heat stress experienced in the weeks preceding the experiment (0.47−4.67 DHW; electronic supplementary material, figure S4). PCA was performed on environmental predictors for the 709 colonies to visualize the distribution of sites and colonies along environmental gradients (electronic supplementary material, figure S3).

### (f) Phenotype by environment analysis

The influence of environmental predictors on heat-tolerance traits (ED50 and performance retention in *F*_v_/*F*_m_ and NDVI) was evaluated using RF ensemble learning and RR on a set of 12 low to moderately correlated predictors (pairwise absolute Pearson correlation = 0.01−0.69; electronic supplementary material, figure S5 and table S8). Because any association of heat-tolerance traits with MMM and DHW at the time of collection may reflect different mechanisms (e.g. adaptation versus acclimatization), both were retained in the dataset despite their strong correlation (*R* = 0.78).

RF models were built using the *cforest* function from the *party* R package [99]. For each of the four heat-tolerance metrics, separate RFs were grown to 1000 trees (ntree) with five environmental predictors tried at each split (mtry). Predictor importance and model accuracy were assessed through repeated 10-fold stratified (across sites) cross-validation (70/30 split; electronic supplementary material, Material and methods). The importance of each predictor was estimated by computing the marginal increase in out-of-bag sample MSE (Mean Inc MSE) when training the model with the predictors randomly shuffled. RR was performed for each phenotypic trait using the *glmnet* R package [100]. The optimal tuning parameter λ of the penalty term was selected using k-fold cross-validation (λ.1se; electronic supplementary material, figures S9 and S10). Model accuracy (*R* ^2^) and environmental predictor coefficients were estimated using bootstrap resampling (repeated 100-fold stratified cross-validation, electronic supplementary material, figure S12 and table S9).

## Data Availability

Data and code are available online [101]. Additional data are provided in the electronic supplementary material [102].
